# Rapid and Simultaneous Prediction of Eight Diesel Quality Parameters through ATR-FTIR Analysis

**DOI:** 10.1155/2018/1795624

**Published:** 2018-02-05

**Authors:** Maurilio Gustavo Nespeca, Rafael Rodrigues Hatanaka, Danilo Luiz Flumignan, José Eduardo de Oliveira

**Affiliations:** ^1^Centro de Monitoramento e Pesquisa da Qualidade de Combustíveis, Biocombustíveis, Petróleo e Derivados (Cempeqc), São Paulo State University (UNESP), R. Prof. Francisco Degni 55 Quitandinha, 14800-900 Araraquara, SP, Brazil; ^2^Instituto Federal de Educação, Ciência e Tecnologia de São Paulo (IFSP), Campus Matão, Rua Estéfano D'avassi, 625 Nova Cidade, 15991-502 Matão, SP, Brazil

## Abstract

Quality assessment of diesel fuel is highly necessary for society, but the costs and time spent are very high while using standard methods. Therefore, this study aimed to develop an analytical method capable of simultaneously determining eight diesel quality parameters (density; flash point; total sulfur content; distillation temperatures at 10% (T10), 50% (T50), and 85% (T85) recovery; cetane index; and biodiesel content) through attenuated total reflection Fourier transform infrared (ATR-FTIR) spectroscopy and the multivariate regression method, partial least square (PLS). For this purpose, the quality parameters of 409 samples were determined using standard methods, and their spectra were acquired in ranges of 4000–650 cm^−1^. The use of the multivariate filters, generalized least squares weighting (GLSW) and orthogonal signal correction (OSC), was evaluated to improve the signal-to-noise ratio of the models. Likewise, four variable selection approaches were tested: manual exclusion, forward interval PLS (FiPLS), backward interval PLS (BiPLS), and genetic algorithm (GA). The multivariate filters and variables selection algorithms generated more fitted and accurate PLS models. According to the validation, the FTIR/PLS models presented accuracy comparable to the reference methods and, therefore, the proposed method can be applied in the diesel routine monitoring to significantly reduce costs and analysis time.

## 1. Introduction

Diesel fuel is a petroleum-derived product of great importance for a country's economy since most of the transportation of industrial and agricultural products depends on diesel vehicles [1, 2]. This fuel is a complex mixture composed mainly of paraffinic, olefinic, and aromatic hydrocarbons ranging from 8 to 28 carbon atoms and, in a lower concentration, substances containing oxygen, nitrogen, sulfur, and metals [3–5]. The diesel composition is influenced by several factors, such as the origin of crude oil, operating variables of the refinery, the addition of fractions from cracking process, and the insertion of additives to increase engine performance [3]. Therefore, the fuel quality is susceptible to many variables until the fuel reaches the consumer. In this perspective, the monitoring of diesel quality parameters is extremely important for commercialization, engine performance, consumer rights, business competition, and environmental risks [5, 6].

The assays performed to ensure the diesel quality are based on standardized procedures that require specific equipment to determine each physicochemical parameter. According to the standard methods, the quality assessment requires considerable sample volume and analysis time, besides the great expense of equipment maintenance and several specialized analysts [7–12]. Therefore, the development of methods to monitor diesel quality accurately, quickly, and environmentally friendly is highly necessary [13]. This becomes possible by attenuated total reflection Fourier transform infrared (ATR-FTIR) spectroscopy associated with multivariate regression methods such as partial least square (PLS). Studies demonstrated the possibility to predict some diesel properties using midinfrared spectroscopy combined with chemometric tools [14–18], some aimed at the prediction of biodiesel content [16], and others were devoted to the identification of diesel adulteration with waste vegetable oils [17, 18].

In USA, European Community, and Japan, the regulations of diesel properties for consumption are established, respectively, by ASTM D975, EN 590, and JIS K2204 [19–21]. In Brazil, the regulation and supervision of fuels are performed according to ANP (National Agency of Petroleum, Natural Gas, and Biofuels) Resolution no. 30/2016, which requires that assays must be conducted according to ASTM, EN, or NBR standards [22]. According to this resolution, at least eight quality parameters of diesel are analyzed in official monitoring laboratories: aspect; color; density; flash point; total sulfur; volatility (distillation temperatures at 10% (T10), 50% (T50), and 85% (T85) recovery); cetane index; and biodiesel content [23].

The development of an alternative method for determining the physicochemical parameters of diesel through ATR-FTIR has several advantages for routine quality monitoring. The use of ATR-FTIR can reduce costs, increase analytical frequency, use smaller sample volume, and provide the determination of all required parameters using only one equipment. Moreover, infrared spectrometers are already purchased by monitoring laboratories for determination of biodiesel content in diesel according to EN 14078.

In view of the high costs and long time required to assess diesel quality by standard methods, this work aimed at the development of a simple and fast analytical method based on ATR-FTIR analysis and PLS regression method to determinate eight diesel quality parameters simultaneously. In this study, multivariate filters and variable selection techniques, such as genetic algorithm (GA), forward interval PLS (FiPLS), and backward interval PLS (BiPLS), were evaluated for the best model predictive ability.

## 2. Materials and Methods

### 2.1. Samples

For eight months, the quality parameters of 3549 samples of diesel fuel were analyzed by Cempeqc (Center for Monitoring and Research of the Quality of Fuels, Biofuels, Crude Oil and Derivatives) according to ASTM and EN standards. The samples were stored at 10°C for further spectroscopic analysis. The standards and equipment used in the determination of quality parameters are presented in Table 1.

Although an extensive sample set can provide greater robustness to a prediction model, this work aimed at the development of a simple method that can be easily reproduced by other laboratories. Therefore, we selected about 10% of the diesel samples for spectroscopic and chemometric analysis. The 3549 diesel samples were divided into groups using hierarchical cluster analysis (HCA) to select the most representative samples. An HCA was executed for each month, and the physicochemical parameters were used as variables. The clusters were performed using 60% of similarity, complete linkage method, and autoscale preprocessing to give the same influence for all variables. The software used for HCA was Pirouette (Infometrix), version 3.11. At the end of the eight months, 409 diesel samples were selected.

### 2.2. Spectroscopic Analysis

The infrared spectra of the 409 samples were obtained by a Nicolet 6700 FTIR spectrometer (Thermo Scientific, Waltham, USA) using 32 scans and 4 cm^−1^ resolution. A Smart ARK ATR sampling accessory of ZnSe crystal and angle of incidence 45° were used to acquire the infrared spectra. The ATR accessory required one milliliter for each sample, and a new background spectrum was acquired every hour to reduce the baseline shifting and ambient variations. The conditions of temperature and relative humidity during the analysis were 20.7 ± 2.0°C and 40 ± 9%, respectively.

### 2.3. Chemometric Analysis

#### 2.3.1. Model Development

The chemometric analysis was executed using Matlab 2013a (MathWorks) with PLS toolbox 7.3.1 (Eigenvector Research Inc.). The FTIR spectra were converted into vectors of 1738 variables and the combination of the vectors resulted in the matrix **X** of dimension 409 by 1738. Prior to the development of PLS models, the sample set was separated into two-thirds for calibration (273 samples) and one-third for validation (136 samples). The Onion algorithm was used to select the samples with less covariance (based on distance from the mean) for each set and, consequently, to obtain greater sample representativeness in both sets [24, 25]. The algorithm was performed for each parameter to ensure that the calibration set had the largest range of reference values.

Initially, the PLS models were developed using the full spectra (full X-block) preprocessed by the mean center or autoscale, depending on the best fit. The number of latent variables (LV) was chosen based on the root mean square errors of calibration (RMSEC), cross-validation (RMSECV), and prediction (RMSEP) in order to minimize the prediction errors and avoid model overfitting [26, 27]. The cross-validation was performed using venetian blinds mode with 10 splits.

Then, statistical tests were applied according to ASTM E1655 [28] to detect the presence of outliers in the calibration and validation sets. Outliers include high leverage samples and samples whose reference values are inconsistent with the model. Therefore, samples with high leverage and studentized residuals were excluded from the sample sets.

#### 2.3.2. Preprocessing Evaluation

Spectral data usually present baseline shifting due to instrumental variations and reflectance deviations [29]. The baseline shifting is typically corrected by applying the first or second derivative, or by polynomials that correct the displacement based on a standard spectrum, for example, multiplicative scatter correction (MSC) and standard normal variate (SNV). In addition, digital filters such as smoothing are also used to improve the signal-to-noise ratio of spectral data [28]. Multivariate filters, such as generalized least squares weighting (GLSW) and orthogonal signal correction (OSC), are less usual preprocesses, but these filters are very useful to eliminate baseline shifting and increase signal-to-noise ratio [30–33]. Therefore, the following preprocessing was evaluated in modeling: mean center, autoscale, Savitzky–Golay smoothing and derivatives, SNV, MSC, GLSW, and OSC.

#### 2.3.3. Variable Selection Methods

Many studies have shown that variable selection is an efficient way to increase the signal-to-noise ratio and, as a consequence, improve the predictive ability of the model [34, 35]. When the noise dominates over the information related to the property of interest, the removal of variables often leads to better accuracy and performance of the analytical method [35, 36]. The selection of variables can be performed based on the spectral knowledge (manual approach) or through algorithms that search for variables that provide the minimum prediction error to the model. Some of the most popular methods for selecting variables are the interval selection method, such as the forward interval PLS (FiPLS), the backward interval PLS (BiPLS), and the genetic algorithm (GA), a technique that employs a probabilistic and nonlocal search process which manipulates binary strings with the coded experimental variables. Details on these variable selection methods can be found in [35].

In this study, four different approaches were evaluated to select variables: manual exclusion, FiPLS, BiPLS, and GA. The manual exclusion was carried out evaluating the spectral residues and loadings plots. Spectral regions with no absorbance or high relative standard deviation (RSD) were excluded from the data and compared with results obtained using the full spectra. Both iPLS methods were executed using interval size of 25 variables, and the number of intervals was determined by the algorithm to obtain the lowest value of RMSECV. The GA was performed with a population size of 128 models, one variable by window, initial terms of 30%, the mutation rate of 0.5%, double crossover, 200 generations, and PLS regression method. All approaches were performed using only the calibration set to avoid overestimated results.

#### 2.3.4. Model Validation

The PLS models were statistically evaluated by figures of merit (FOM) according to ASTM E1655 and Valderrama et al. [28, 37]. The accuracy of the models, defined as the degree of agreement between a measured value and reference value, was assessed by the values of RMSECV, RMSEP, correlation coefficients (*r*), average relative errors (ARE), and relative percent difference (RPD). The RMSECV was obtained by cross-validation using the venetian blinds mode with 10 splits, and the RMSEP was obtained by the validation samples that were measured independently from the calibration samples. Then, the RMSECV and RMSEP were compared with the reproducibility of the reference method. The ARE was used as a parameter to evaluate the magnitude of the prediction errors in relation to the reference values [38]. The ARE value was calculated by

(1) $$\begin{array}{c} \text{ARE}\left(\%\right)=\frac{\sum_{i=1}^{n_{v}}{\left({\hat{y}}_{i}-y_{i}/y_{i}\right)}^{2}}{n_{v}}\times 100, \end{array}$$

where *y*_*i*_ and ${\hat{y}}_{i}$ correspond, respectively, to the reference value and predicted value by the model and *n*_*v*_ is the number of validation samples. The relative percent difference (RPD) was obtained by the ratio of the standard deviation of the validation set reference values to the RMSEP value. RPD values above 2.5 indicate that the model has acceptable accuracy over the measurement range, while values above 10 are considered excellent for alternative methods [39].

Linearity is an important parameter to evaluate the performance of the model since the PLS regression method is not suitable for nonlinear relationships between the variables *x* and the property of interest [40]. The linearity corresponds to the ability of the model to provide results directly proportional to the property of interest. One way to evaluate this parameter in multivariate models is through the residues of calibration and validation samples plots. If the distribution of residues is random, it can be said that the model shows a linear behavior. In addition to the residue plots, the linearity was also evaluated by the values of determination coefficients (*R* ^2^) and bias. This last FOM indicates the presence of systematic errors in the model. Bias can be assessed by a *t*-test for the validation samples at a confidence interval of 95%. The average bias was calculated by summing the differences between the reference value and the predicted value divided by the number of validation samples [28]:

(2) $$\begin{array}{c} \text{bias}=\frac{\sum_{i=1}^{n_{v}}{\left(y_{i}-{\hat{y}}_{i}\right)}^{2}}{n_{v}}. \end{array}$$

Then, the standard deviation of validation errors (SDV) was calculated as

(3) $$\begin{array}{c} \text{SDV}=\sqrt{\frac{\sum {\left[\left(y_{i}-{\hat{y}}_{i}\right)-bias\right]}^{2}}{n_{v}-1}}, \end{array}$$

and finally, the value of *t*_bias_ was given by

(4) $$\begin{array}{c} t_{\text{bias}}=\frac{\left|\text{bias}\right|\sqrt{n_{v}}}{\text{SDV}}. \end{array}$$

If the value obtained for *t*_bias_ was greater than the critical value for *n*_*v*_ − 1 degrees of freedom, then the multivariate model presented significant systematic errors.

The precision of the models was evaluated by the analysis of 14 replicates of 30 diesel samples performed on different days. The average of relative standard deviations (RSD) and the intermediate precision—calculated through (5), where *n* is the number of samples and *m* the number of replicates—were used as parameters [37]. Then, the intermediate precision was compared to the repeatability value of the reference method:

(5) $$\begin{array}{c} \text{intermediate precision}=\sqrt{\frac{\sum_{i=1}^{n}\sum_{j=1}^{m}{\left(y_{i}-{\hat{y}}_{i}\right)}^{2}}{n\left(m-1\right)}}. \end{array}$$

## 3. Results and Discussion

### 3.1. Physicochemical Assays

The values of reproducibility and repeatability of the reference methods, the range of measured values of each quality parameter, and the number of samples in nonconformity with ANP Resolution no. 65 [41] are shown in Table 2. The quality parameter that presented the highest number of nonconforming samples was T10, followed by T85 and biodiesel content. As ANP Resolution no. 65 allows only a variation of 0.5% (*v*/*v*) of biodiesel content, most of the samples were in a narrow range of concentration. The same occurred with the total sulfur but in two different ranges of concentration due to the availability of two types of commercial diesel with distinct sulfur content.

### 3.2. Spectroscopic Analysis

**Figure 1:**
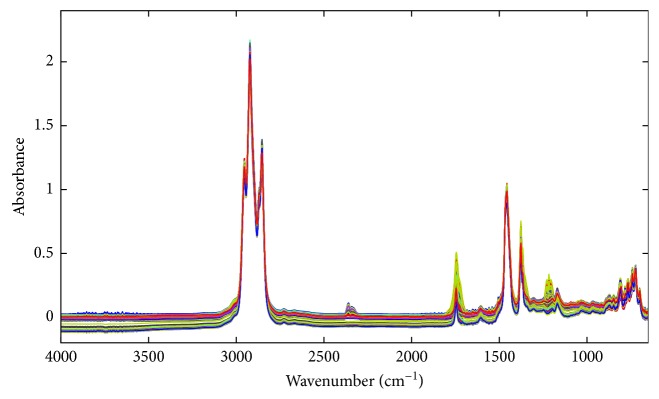
Infrared spectra of all diesel samples.

The FTIR spectra of all diesel samples are represented in Figure 1. Functional groups of the constituents of samples could be observed by characteristic absorption bands of each group of atoms through the infrared spectra.

The most intense bands were caused by C–H groups stretch (3000–2800 cm^−1^) and angular deformations (1464 cm^−1^ and 1379 cm^−1^) [42]. The bands at 2350 cm^−1^ and 667 cm^−1^ were, respectively, results of asymmetrical stretch and angular deformation of CO_2_ molecules present in the atmosphere [43]. The presence of biodiesel in the samples was observed by carbonyl absorption band (1750–1735 cm^−1^) and aliphatic ester absorption band (1300–1000 cm^−1^). Aromatic compounds had characteristic bands of low intensity in 900–675 cm^−1^ from the C–H out-of-plane angular deformation. The sulfur is present in diesel as mercaptans and sulfides, and it was observed by S–H axial stretch at 2600–2550 cm^−1^ and C–S axial stretch at 700–650 cm^−1^ [43, 44]. The S–H stretch was very weak; however, few groups have absorption in this region, so it was useful for the total sulfur parameter. The vibrational group attribution to each band is present in Table 3.

### 3.3. Chemometric Analysis

#### 3.3.1. Outlier Detection

During calibration, outlier statistics were applied to identify samples that had unusual leverage and studentized residuals. The outlier detection was performed prior to the variable selection because the exclusion of variables may reduce outlier detection capabilities of the model [28]. The number of outliers from each sample set is shown in Table 4. Considering the calibration and validation sample set with, respectively, 273 and 136 samples, the number of outliers (3% maximum) was not significant for the prediction models.

High studentized residual values may be the result of errors in the reference measurement, spectral acquisition error, reference value transcription, or even a failure of the model. Error in the spectral acquisition would lead to the presence of the same outlier in all models of prediction; however, different outliers were detected for each model. The absence of new outliers in the model after removal of the anomalous samples indicated that there was no failure in the model. Therefore, errors in the reference values were most likely responsible for the presence of outliers.

#### 3.3.2. Preprocessing Evaluation

The baseline shifting in the raw spectra was observed in Figure 1. The shifting may be the result of variations in the position of the ZnSe crystal since it was removed from the spectrometer for cleaning before each analysis. All the evaluated preprocessing—derivatives, MSC, SNV, GLSW, and OSC—provided baseline correction and higher correlation coefficients than mean center or autoscale preprocessing. Moreover, multivariate filters (GLSW and OSC) provided models with greater explained variance using fewer latent variables (Table 5). Therefore, all models were preprocessed using OSC, except the model for T85, which presented better fit with GLSW preprocessing.

#### 3.3.3. Variable Selection

**Figure 2:**
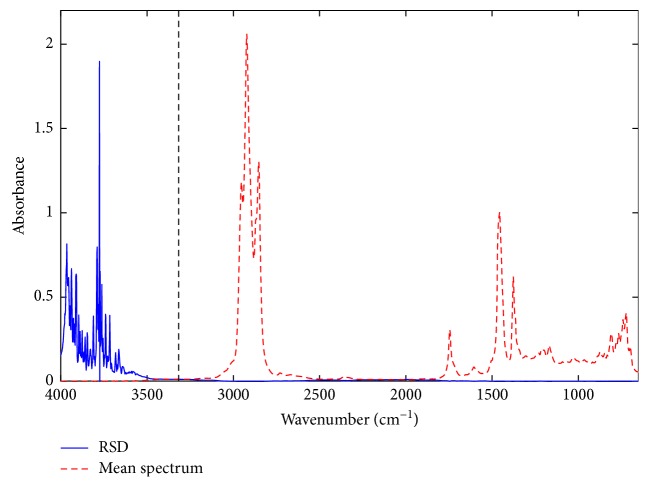
The mean spectrum (red line) and relative standard deviation (blue line) of 14 replicates of a diesel sample.

The exclusion of regions without information of sample constituents or low signal-to-noise ratio may improve the performance of the models [35]. In Figure 2, the noisy spectral regions can be observed through the relative standard deviation (RSD), represented by the blue line, and calculated from the mean of 14 replicates, represented by the red line. In addition, there was no absorption by the components of diesel in the ranges 4000–3100 cm^−1^ and 2450–1950 cm^−1^; thus, these spectral regions were excluded, and new models were developed.

The RMSEP and correlation coefficient of validation (*r*_val_) obtained by the different variable selection approaches are presented in Table 6. The manual exclusion of variables provided better results only for the prediction models of flash point, T10, cetane index, and biodiesel content. The manual selection of variables had the risk of inadvertent exclusion of important variables for the modeling, impairing the performance of the model.

The selection of variables by interval selection methods reduces the values of RMSEC and RMSECV but might decrease the predictive ability of the model. The FiPLS method usually uses few intervals to correlate the spectral variables with the property of interest and, as consequence, the calibration model is more susceptible to overfitting and the prediction of unknown samples is impaired, especially properties that are correlated to several spectral variables. As the sulfur content is correlated only to the S–H and C–H bond variables, the FiPLS method provided the best fit to the model.

The distillation temperatures and the cetane index depend on the size and structure of the hydrocarbon chains of the diesel components; therefore, these are properties related to several functional groups with response in the midinfrared region. Thus, the selection methods such as BiPLS, which seek to exclude noisy variables rather than including variables more correlated to the property of interest, tend to be more suitable for optimization of these diesel parameters.

The analytical signals in the midinfrared region result in many correlated variables; that is, FTIR data present many collinearities. Normally, the problem of collinearity can be attenuated by the application of the genetic algorithm, since the spectral variables are manipulated in binary strings and the search for variables that provide a minor error of prediction is performed by a probabilistic and nonlocal process [35]. GA was the best variable selection approach for prediction models of density, flash point, T50, and biodiesel content.

In general, the selection of variables by iPLS and GA provided improvements in the predictive ability of the calibration models, except for T85, and the difference between the results obtained by both algorithms was not significant. The selected variables used in the best-fitted models are presented in the supplementary material (available here) attached to the article.

#### 3.3.4. Model Performance

After defining the most appropriate variable selection method for each parameter, the figures of merit were determined for the prediction models (Table 7). The complexity of the diesel composition, consisting of hundreds of compounds, generates a large amount of information in the FTIR spectra and, therefore, the correlation between the matrix **X** and the property of interest requires a considerable number of LVs. Although the use of OSC reduces the collinearity problem and increases the captured variance of the **X** and **y** blocks, several analytical signals were correlated to the properties of diesel, so several LVs were required.

**Figure 3:**
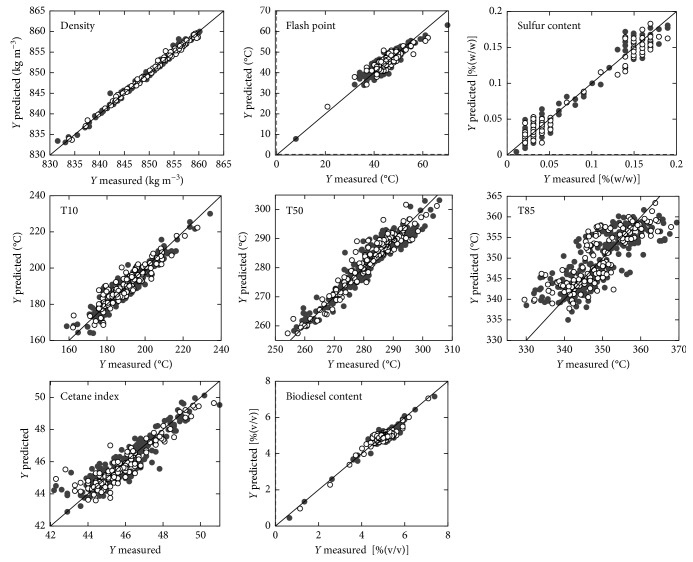
Predicted versus measured value plots of the PLS models.

The accuracy of the models was evaluated by comparing the RMSEP values (Table 7) with the reproducibility values of the reference methods (Table 2). Since all models presented RMSEP values below or equivalent to the reproducibility value, the FTIR/PLS method could be considered accurate for predicting diesel parameters. In addition, the correlation coefficients were above 0.89, except for T85; thus, the predicted values were well correlated with the reference values (Figure 3).

Although the prediction model for sulfur content presented *r*_val_ equal to 0.987, the obtained ARE value was high when compared to the others. The high relative errors that resulted in ARE equal to 14.10% were caused by the low sensitivity of the model for prediction of S500 diesel samples. However, the RPD value indicated that the model was accurate when the RMSEP value is compared to the sulfur content range of the validation sample set.

The determination coefficients (*R* ^2^) indicated that the prediction models of flash point and T85 presented lower linearity than the other parameters. Figure 3 shows that, for these parameters, the residues tend to be negative values with the increase of the reference value. Although the models have low bias values, the *t-*test revealed that there were systematic errors in the prediction models for sulfur content, T85, and biodiesel content. Since the models for sulfur and biodiesel content presented good linearity (*R* ^2^_val_ > 0.88), the presence of systematic errors can be reduced by the addition of more samples to the model.

The precision of the models was evaluated by the analysis of 30 diesel samples on 14 consecutive days. Although the samples were stored at 10°C between the analyses, the diesel fuel consists of semivolatile compounds and, therefore, changes in sample composition during the replicates acquisition imply an increase in measurement uncertainty. The intermediate precision values of the models were above the repeatability values of the reference methods, except for the biodiesel content prediction. However, the RSD values showed that almost all models had good precision (RSD below 1%) and only the models for prediction of flash point and sulfur content presented low precision.

Although the prediction models for flash point, sulfur content, and T85 have the limitations mentioned above, the conformity ranges of these parameters (Table 2) can be met by FTIR/PLS models with reliability since the accuracy and precision of the method are known. If an unknown sample is analyzed by FTIR and the result obtained is in the nonconformity range, it is recommended that the result is confirmed by the standard method. Since only about 2% of the diesel samples in Brazil presented nonconformities in 2017 [45], the FTIR/PLS method can be applied in routine monitoring of diesel quality to reduce the costs and time of analysis.

## 4. Conclusions

This study showed the possibility of applying ATR-FTIR spectroscopy with PLS regression method to predict the quality parameters (density; flash point; total sulfur content; distillation temperatures at 10% (T10), 50% (T50), and 85% (T85) recovery; cetane index; and biodiesel content) in commercial diesel samples.

All the evaluated preprocessing (derivatives, MSC, SNV, GLSW, and OSC) provided baseline correction and higher correlation coefficients. In addition, the GLSW and OSC preprocessing provided greater explained variance to the model using fewer latent variables. The selection of variables by iPLS or GA provided better predictive ability to the calibration models, except for T85. However, the difference between the results obtained by both algorithms was not significant.

According to the model validation, all PLS models presented acceptable accuracy when compared to the values of reproducibility and had good precision, except for sulfur content prediction of S500 diesel samples. Since the application of the ATR-FTIR/PLS method is able to reduce costs and increase considerably the analytical frequency, the diesel quality monitoring programs, as well as the final consumer, can benefit greatly from the application of the proposed method.

## Figures and Tables

**Table 1:** Standard methods and equipment used to determine the quality parameters of diesel samples.

Diesel property	Method	Equipment
Density	ASTM D4052	DMA 4500 automatic densimeter (Anton Paar)
Flash point	ASTM D93	PMA-4 flash point automatic analyzer (Petrotest)
Total sulfur	ASTM D4294	EDX-800 spectrometer (Shimadzu)
Distillation	ASTM D86	AD-6 automatic atmospheric distiller (Tanaka)
Cetane index	ASTM D4737	—
Biodiesel content	EN 14078	Nicolet IR200 spectrometer (Thermo Scientific)

**Table 2:** Results of quality parameter assays using the reference methods in accordance with ANP Resolution no. 65 [41].

Quality parameter	Unit	Reproducibility	Repeatability	Range of conformity	Range of measured values	Number of nonconforming samples
Density	kg·m^−3^	±0.5	±0.1	820.0 to 865.0	830.6 to 860.4	0
Flash point	°C	±3	±1	38	8 to 70	16
Total sulfur	% (*w*/*w*)	±0.01	±0.002	<0.05^a^ or < 0.18^b^	0.01 to 0.19	4
T10	°C	±4.2	±1.8	>180.0	158.9 to 234.1	75
T50	°C	±3.0	±0.9	245.0 to 310.0	250.9 to 305.8	0
T85	°C	±5.2	±1.4	>360.0	247.3 to 369.5	40
Cetane index	—	±2.0	—	>42.0	41.1 to 54.2	1
Biodiesel content	% (*v*/*v*)	±0.2	±0.1	4.5–5.5	0.6 to 7.4	41

^a^For S50 diesel samples, maximum sulfur content is 0.05% (*w*/*w*); ^b^for S1800 diesel samples, maximum sulfur content is 0.18% (*w*/*w*).

**Table 3:** Infrared vibrational groups of the diesel samples [42, 43].

Attribution	Wavenumber (cm^−1^)
CH_3_ asymmetrical stretch	2953
CH_3_ symmetric stretch	2870
CH_3_ angular deformation	1379
CH_2_ asymmetric stretch	2922
CH_2_ symmetrical stretch	2853
CH_2_ angular deformation	1464
CO_2_ asymmetrical stretch	2350
CO_2_ angular deformation	667
C=O carbonyl stretch	1750–1735
C–O stretch (aliphatic ester)	1300–1000
C=C stretch (alkenes)	1660–1600
C=C stretch (aromatic)	1600 and 1475
=C–H stretch (aromatic)	900–690
S–H stretch	2600–2550
C–S stretch	700–600

**Table 4:** The number of outliers removed from the calibration and validation sets.

	Density	Flash point	Total sulfur	T10	T50	T85	Cetane index	Biodiesel content
Calibration (273 samples)	2 (1%)	1 (1%)	2 (1%)	3 (1%)	3 (1%)	4 (2%)	3 (1%)	0
Validation (136 samples)	3 (2%)	1 (1%)	2 (2%)	4 (3%)	2 (2%)	1 (1%)	3 (2%)	0

**Table 5:** Comparison between mean center/autoscale and multivariate filters.

	Mean center/autoscale^∗^					Multivariate filter				
	Number of LVs	RMSEP	*r* (val)	X-block variance (%)	y-block variance (%)	Number of LVs	RMSEP	*r* (val)	X-block variance (%)	y-block variance (%)
Density	12	0.5	0.9959	99.76	99.17	9	0.5	0.9957	99.99	99.14
Flash point	12	2.3	0.8735	99.72	83.78	8	2.3	0.8761	99.99	83.20
Total sulfur	7	0.013	0.9773	99.31	94.52	6	0.013	0.9806	99.99	95.05
T10	11	4.3	0.9192	99.69	86.90	9	4.2	0.9224	99.99	88.04
T50	7	3.5	0.9432	99.43	86.18	6	3.2	0.9508	89.92	89.47
T85	11	4.8	0.7909	99.67	70.31	8	4.7	0.7991	99.98	66.18
Cetane index	10	0.6	0.9084	99.63	83.02	9	0.6	0.9103	99.99	83.78
Biodiesel content	10	0.2	0.9337	99.71	88.50	5	0.2	0.9302	97.13	89.85

^∗^The models for density, cetane index, and biodiesel content were preprocessed using mean centering.

**Table 6:** PLS models using different variable selection approaches.

Variable selection	Number of variables	Number of LVs	RMSEP	*r* (val)	Number of variables	Number of LVs	RMSEP	*r* (val)
	Density				Flash point			
None	1738	9	0.50	0.996	1738	8	2.30	0.876
Manual	1124	10	0.55	0.995	1124	7	2.20	0.888
FiPLS	225	8	0.46	0.997	300	6	2.23	0.885
BiPLS	1363	8	0.49	0.996	1663	8	2.19	0.889
GA	439	9	0.43	0.997	380	7	2.17	0.889
	*Sulfur content*				*T10*			
None	1738	6	0.013	0.981	1738	9	4.19	0.922
Manual	1124	6	0.014	0.976	1124	10	4.16	0.923
FiPLS	175	5	0.011	0.987	350	8	4.24	0.920
BiPLS	1363	7	0.011	0.985	1563	10	4.13	0.925
GA	434	7	0.011	0.986	370	9	4.24	0.921
	*T50*				*T85*			
None	1738	6	3.20	0.951	1738	8	4.70	0.799
Manual	1124	5	3.24	0.951	1124	8	4.78	0.793
FiPLS	75	8	3.26	0.949	75	6	4.90	0.775
BiPLS	1638	6	3.10	0.954	1088	10	4.75	0.797
GA	410	5	2.97	0.957	395	6	4.81	0.788
	*Cetane index*				*Biodiesel content*			
None	1738	9	0.64	0.910	1738	5	0.21	0.930
Manual	1124	8	0.63	0.912	1124	9	0.20	0.936
FiPLS	225	7	0.62	0.914	150	8	0.21	0.932
BiPLS	1363	7	0.61	0.917	873	8	0.20	0.935
GA	405	6	0.62	0.914	452	6	0.20	0.937

**Table 7:** Validation results of prediction models for quality parameters of diesel.

	Density	Flash point	Sulfur content	T10	T50	T85	Cetane index	Biodiesel content
Unit	kg·m^−3^	°C	% (*w*/*w*)	°C	°C	°C	—	% (*v*/*v*)
Measured interval	831.6 to 860.4	8 to 70	0.01 to 0.19	158.9 to 234.1	256.3 to 305.8	330 to 369.5	42.2 to 51.0	0.6 to 7.4
Variable selection	GA	GA	FiPLS	BiPLS	GA	None	BiPLS	GA
Number of variables	439	380	175	1563	410	1738	1363	452
Number of LVs	9	7	5	10	5	8	7	6
Accuracy								
RMSEC	0.4	2	0.01	3.9	3.0	4.6	0.6	0.2
RMSECV	0.5	2	0.01	4.4	3.4	5.1	0.6	0.2
RMSEP	0.4	2	0.01	4.1	3.0	4.7	0.6	0.2
*r* (cal)	0.997	0.930	0.987	0.940	0.954	0.814	0.919	0.946
*r* (CV)	0.996	0.900	0.984	0.922	0.941	0.770	0.903	0.936
*r* (val)	0.997	0.889	0.987	0.925	0.957	0.799	0.917	0.937
ARE (%)	0.04	3.72	14.10	1.68	0.77	1.06	0.95	3.18
RPD	12.89	2.19	5.93	2.63	3.44	1.64	2.49	2.83
Linearity								
*R* ^2^ cal	0.994	0.866	0.974	0.883	0.909	0.662	0.845	0.894
*R* ^2^ CV	0.993	0.809	0.969	0.851	0.886	0.593	0.815	0.877
*R* ^2^ val	0.994	0.791	0.974	0.855	0.916	0.639	0.841	0.878
Bias	0.002	0.049	−0.002	0.325	0.340	0.822	−0.086	−0.037
*t*_bias_	0.05	0.26	2.20	0.91	1.33	2.06	1.66	2.16
Intermediary precision	1.5	2	0.01	1.9	1.5	2.1	0.4	0.1
RSD (%)	0.04	5.91	9.00	0.93	0.44	0.47	0.40	0.95

^∗^ *t* critical (95% confidence level; 135 degrees of freedom) = 1.98.

## Supplementary Materials

Supplementary files In the supplementary material can be visualized the variables used in the PLS models that presented better predictive abilities. The variable selection method used for each diesel property is also presented in the supplementary material. Supplementary Figure 1: spectra variables of GA-PLS model for density prediction. Supplementary Figure 2: spectra variables of GA-PLS model for flash point prediction. Supplementary Figure 3: spectra variables of FiPLS model for sulfur content prediction. Supplementary Figure 4: spectra variables of BiPLS model for T10 prediction. Supplementary Figure 5: spectra variables of GA-PLS model for T50 prediction. Supplementary Figure 6: spectra variables of PLS model for T85 prediction. Supplementary Figure 7: spectra variables of BiPLS model for cetane index prediction. Supplementary Figure 8: spectra variables of GA-PLS model for biodiesel content prediction.
