# Supplementary material for: Rapid and Simultaneous Prediction of Eight Diesel Quality Parameters through ATR-FTIR Analysis
Source: J Anal Methods Chem. 2018 Feb 5;2018:1795624. doi: 10.1155/2018/1795624 (PMC5832161; doi:10.1155/2018/1795624)
Supplement: Supplementary files — In the supplementary material can be visualized the variables used in the PLS models that presented better predictive abilities. The variable selection method used for each diesel property is also presented in the supplementary material. Supplementary Figure 1: spectra variables of GA-PLS model for density prediction. Supplementary Figure 2: spectra variables of GA-PLS model for flash point prediction. Supplementary Figure 3: spectra variables of FiPLS model for sulfur content prediction. Supplementary Figure 4: spectra variables of BiPLS model for T10 prediction. Supplementary Figure 5: spectra variables of GA-PLS model for T50 prediction. Supplementary Figure 6: spectra variables of PLS model for T85 prediction. Supplementary Figure 7: spectra variables of BiPLS model for cetane index prediction. Supplementary Figure 8: spectra variables of GA-PLS model for biodiesel content prediction. [file 1795624.f1.pdf]

SUPPLEMENTARY MATERIAL

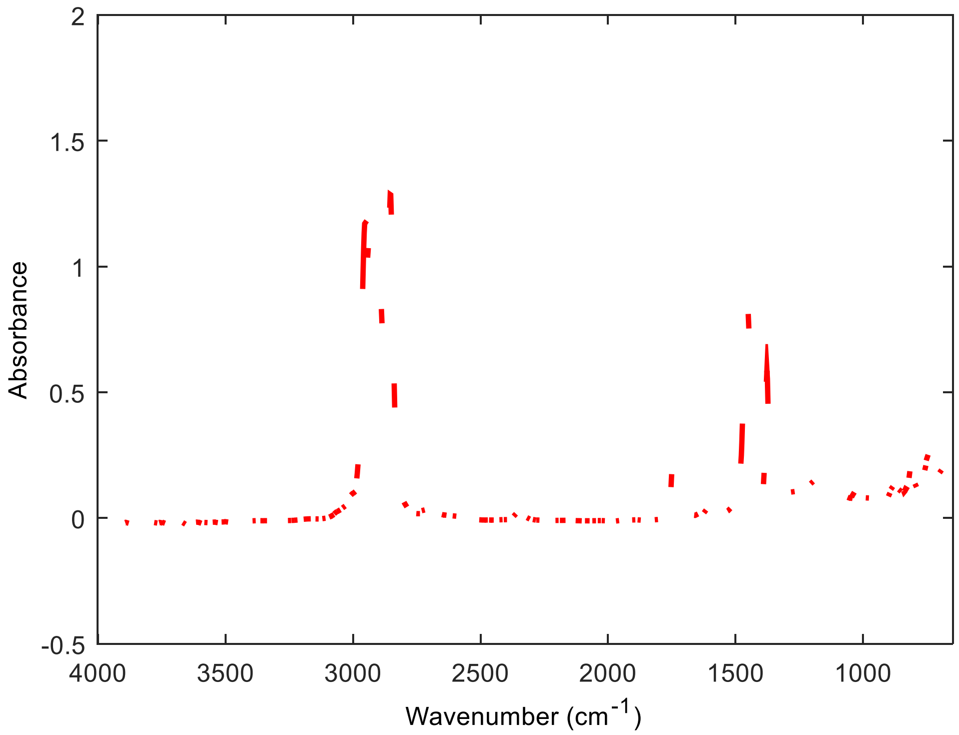

Figure 1. Spectra variables of GA-PLS model for density prediction.

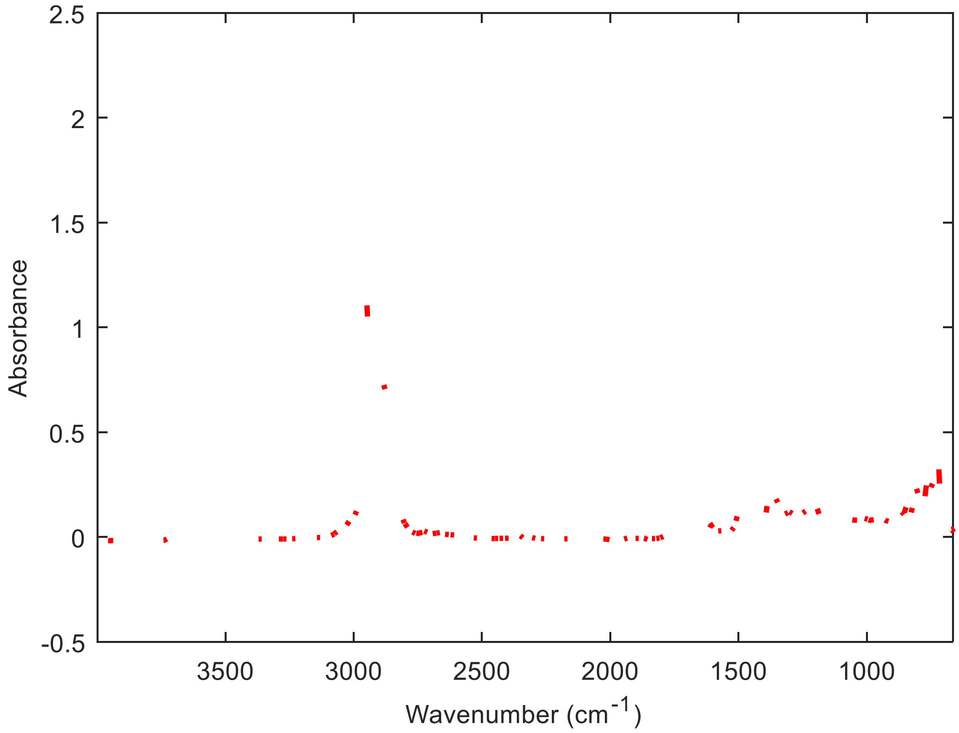

Figure 2. Spectra variables of GA-PLS model for flash point prediction.

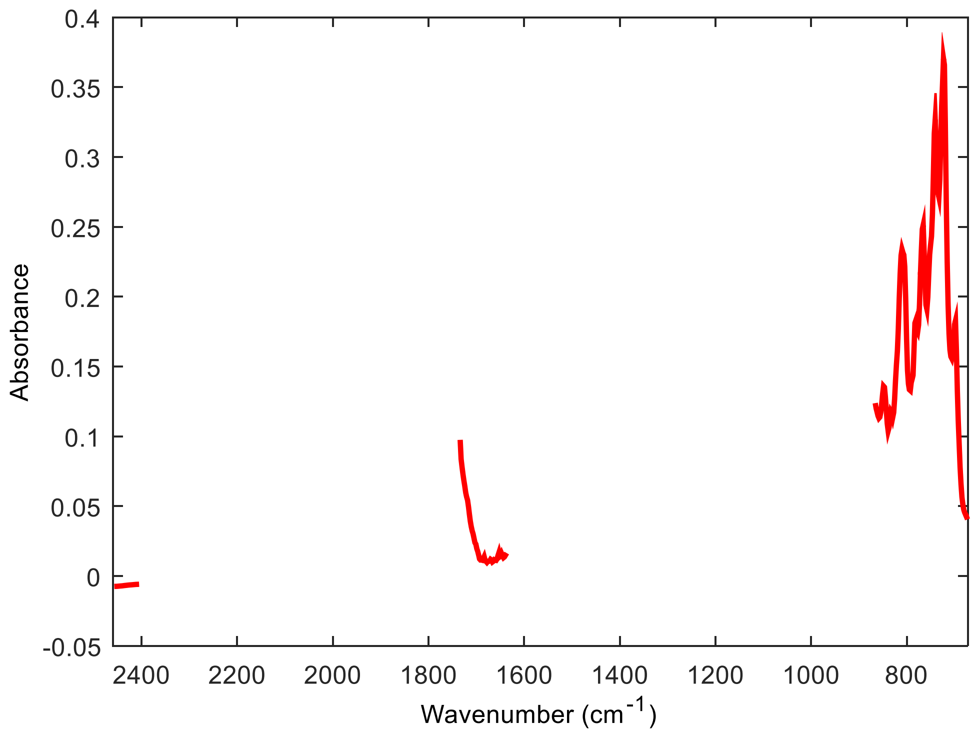

Figure 3. Spectra variables of FiPLS model for sulfur content prediction.

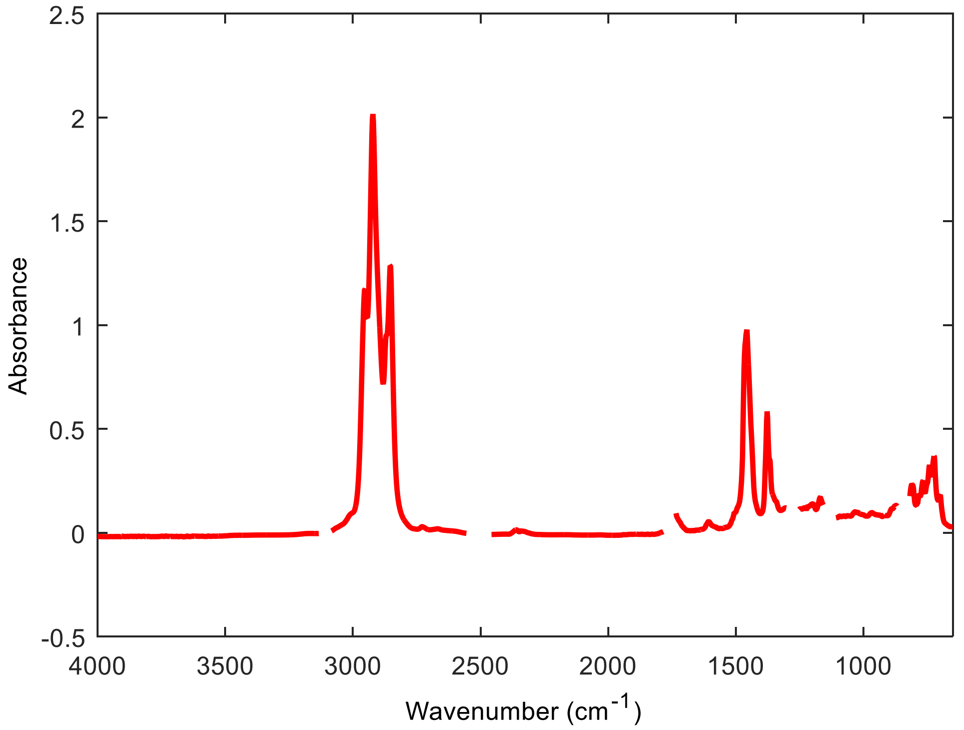

Figure 4. Spectra variables of BiPLS model for T10 prediction.

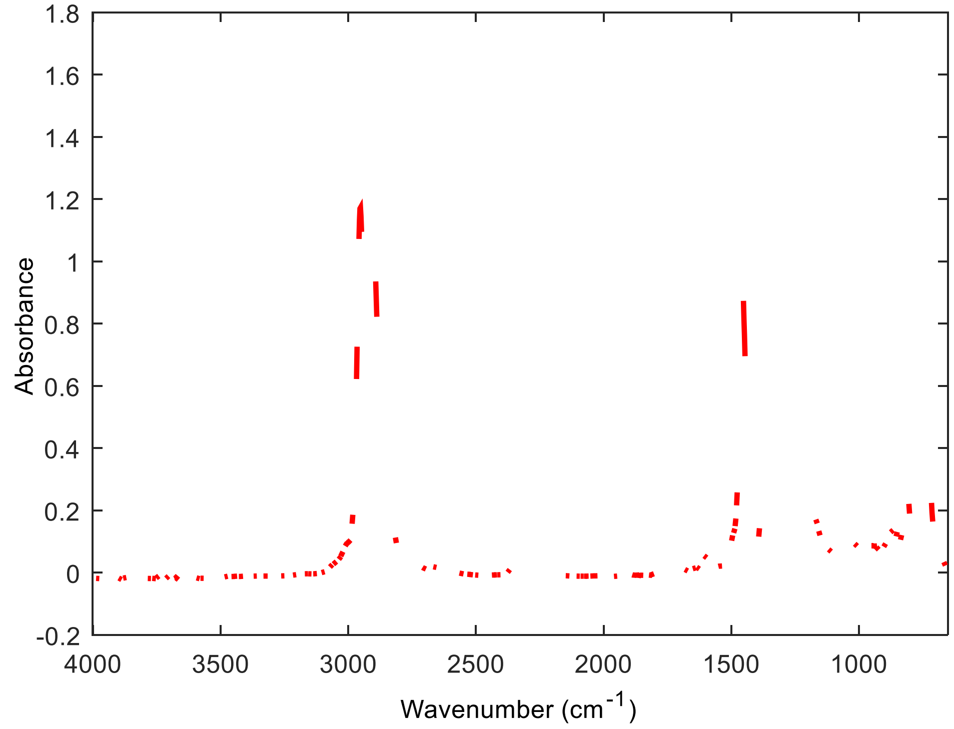

Figure 5. Spectra variables of GA-PLS model for T50 prediction.

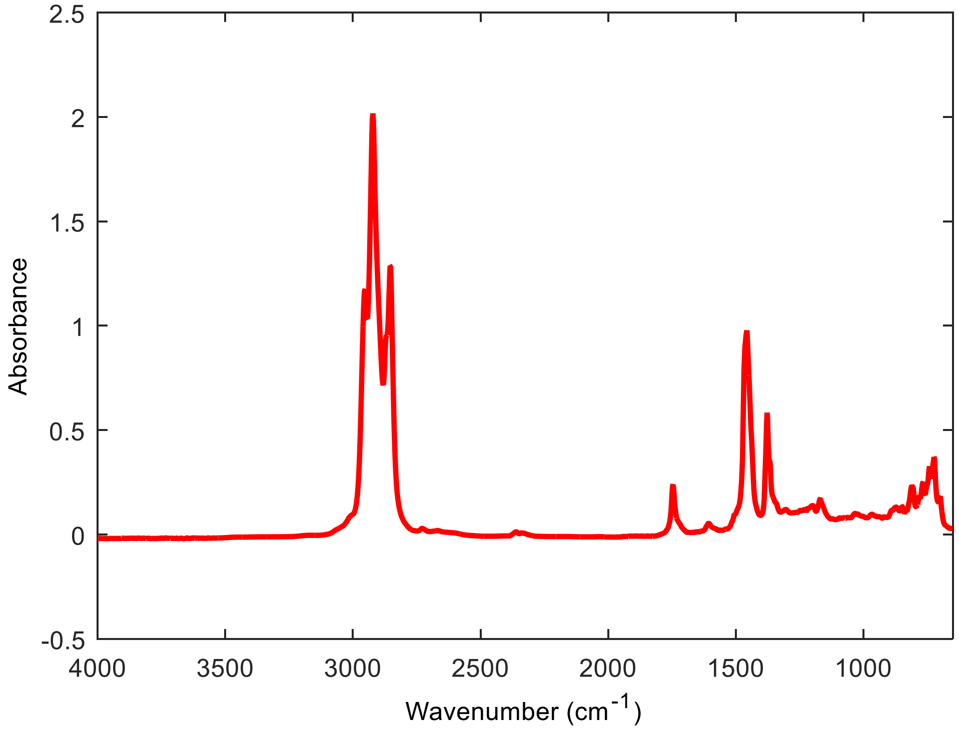

Figure 6. Spectra variables of PLS model for T85 prediction

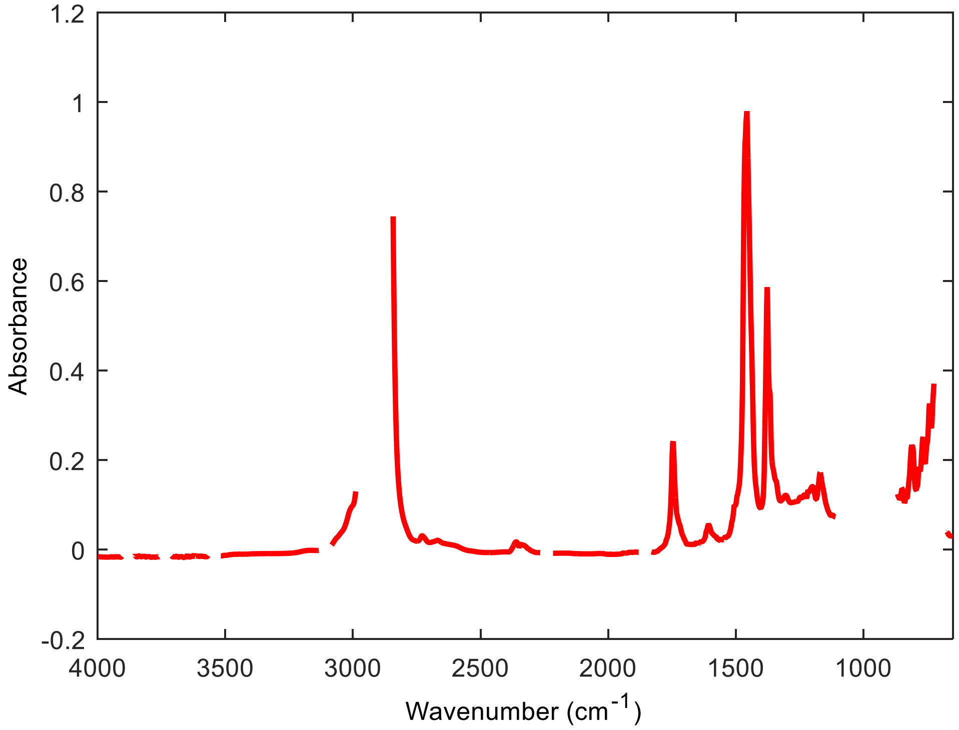

Figure 7. Spectra variables of BiPLS model for cetane index prediction.

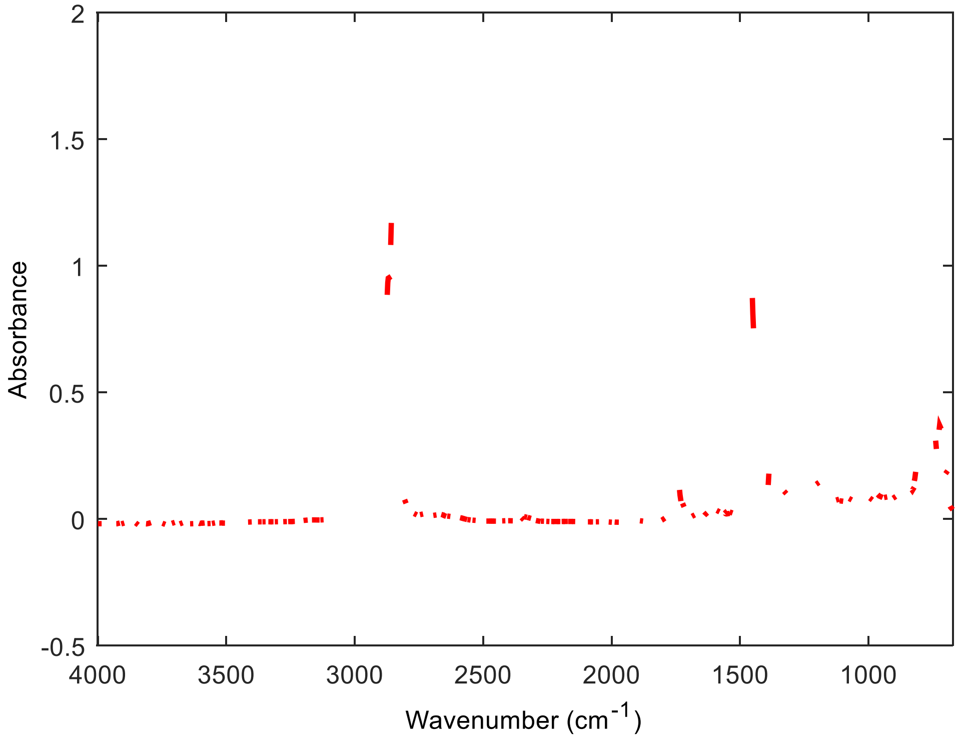

Figure 8. Spectra variables of GA-PLS model for biodiesel content prediction.
